# Effect of different head-high lateral extubation on adverse reactions in the peri-extubation period of pediatric OSAS surgery under general anesthesia

**DOI:** 10.1186/s12871-023-02099-9

**Published:** 2023-04-27

**Authors:** Yun Zhou, Zhonglan Lin, Xinlei Lu, Yaqin Huang, Weiping Lei, Jianliang Sun

**Affiliations:** 1 Department of Anesthesiology, Fourth Clinical School of Medicine, Zhejiang Chinese Medical University, Hangzhou, 310000 China; 2 Department of Anesthesiology, Affiliated Hangzhou First People’s Hospital, Zhejiang University School of Medicine, Hangzhou, 310000 China

**Keywords:** Pediatric, General anesthesia, OASA, Airway complications, Position of the body

## Abstract

**Background:**

Children with OSAS are prone to various airway complications during tracheal extubation after tonsillectomy and adenoidectomy due to oropharyngeal secretions and oozing blood. However, few studies have examined the effect of position on airway complications after tracheal extubation in children with OSAS. The aim of this study was to investigate the appropriate position for extubation in children with OASA.

**Methods:**

A total of 459 children aged 3–14 years with OSAS who underwent tonsillectomy and adenoidectomy were recruited for this study. All children were treated with the same surgical approach and standard anesthesia methods of induction of anesthesia, tracheal intubation and maintenance of anesthesia. At the end of surgery, the children were delivered to the post anesthesia care unit and randomly divided into three groups: Group A: Head-high 0° in lateral position; Group B: Head-high 15° in lateral position; Group C: Head-high 30° in lateral position. The main outcomes of this study were the pulse oxygen saturation (SpO_2_) and the Sedation-Agitation Scale (SAS) scores of the children after extubation, the outflow of oral-nasal secretions and the respiratory complications. Secondary outcomes were blood pressure, heart rate, end-respiratory carbon dioxide, respiratory rate, and post-operative awakening time of the children in three groups.

**Results:**

Data from a total of 423 children were statistically analyzed, 141 in Group A, 142 in Group B, and 140 in Group C. The main results showed a significant decrease in choking response after extubation in Group B (46.5%) and Group C (40.7%) compared to Group A (60.3%) (*P* < 0.05). The SAS score for postoperative agitation was higher in Group A (4.6 $\pm$ 0.9) than in Group B (4.4 $\pm$ 0.7) and Group C (4.3 $\pm$ 0.6) (*P* < 0.05). Also the SpO_2_ after extubation was higher in Group B (97.2%) and Group C (97.1%) than in Group A (95.8%) (*P* < 0.05). In contrast, there was no difference in the occurrence of respiratory complication and postoperative agitation in children between Group B and Group C (all *P* > 0.05). In addition, there was no difference in the amount of oral-nasal secretions among the children in the three groups (all *P* > 0.05).

**Conclusion:**

The head-high 15° lateral position and head-high 30° lateral position can reduce the incidence of airway complications and agitation and provide safe and comfortable extubation conditions for children during the peri-extubation period after tonsillectomy and adenoidectomy, which has certain clinical guidance value.

**Trial registration:**

Registration Number: NO.ChiCTR2200055835(20,01,2022) https://www.chictr.org.cn

## Introduction

The most common disorder of sleep apnea in children is obstructive sleep apnea syndrome (OSAS) [1], characterized by interrupted breathing and partial or complete obstruction of the upper airway, the main factor is the enlargement of the tonsils and adenoids in the pediatric pharynx. The clinical symptoms are snoring, and open-mouth breathing, then resulting in hypoventilation and hypoxemia [1–4]. Due to the age of children, tonsillectomy and adenoidectomy are often performed under general anesthesia with tracheal intubation [5–7], but residual anesthetic drugs during the awakening period often inhibit respiratory and defensive functions (e.g., cough reflex) in children to varying degrees. However, once the children have excessive respiratory secretion, it is easy to cause laryngospasm, airway obstruction and other respiratory complications, leading to serious consequences such as hypoxemia and asphyxia, which also affect the effect of surgical treatment and early recovery of the children [8].

It is accustomed to perform tracheal extubation with the supine position during the awakening period of general anesthesia in children, which may be related to the familiarity of doctors and nurses with the supine operation. However, compared with the supine position, extubation in the lateral position can improve the patency of the airway in children after tonsillectomy and adenoidectomy [9] and facilitates spontaneous breathing in children after surgery. It was found that the lateral position during the awakening period significantly reduced the choking response and the degree of agitation after extubation. Thomas found that the risk of hypoxemia and the degree of oxygen saturation reduction are bigger when the mask was removed in the supine position, and the most serious complication also occurred in the supine position [10]. Therefore, he concluded that the lateral position is preferable to the supine position for mask removal. However, it has been observed that lateral position extubation at the conventional level (i.e., head-high 0°) may increase the risk of gastric contents regurgitation [11], whereas head-high extubation prevents regurgitation of gastric contents. It has also been shown that patients with general anesthesia are more comfortable in an appropriate head-high position (15° to 30°) after awakening. In addition, head-high position can significantly reduce the choking response, which in turn avoids the adverse effects of the sudden rise in intracranial and thoracic pressure caused by choking, such as headache, nausea, vomiting, and breathing difficulties [12].

There are few reports on the effect of different positions on complications related to tracheal extubation after tonsillectomy and adenoidectomy under general anesthesia in children with OSAS. Previous studies have demonstrated that lateral position or head-high position can improve airway patency in children after tonsillectomy and adenoidectomy, but there is no detailed report on whether the head-high lateral position is a better position for extubation after tonsillectomy and adenoidectomy and the appropriate height of head-high position is not clear and definite. In this study, we proposed to use different height of head-high lateral position to observe and compare the occurrence of choking, agitation, decreased pulse oxygen saturation (SpO_2_) and the oral and nasal secretions during the awakening period, and we aimed to explore the appropriate position for tracheal extubation in children with OSAS during the awakening period after tonsillectomy and adenoidectomy.

## Materials and methods

### Study design

The study was approved by the Medical Ethics Committee of the Hangzhou First People's Hospital, affiliated with Zhejiang University, with ethical approval number IIT-20220217–0022, and registration was completed at the China Clinical Trials Registry with registration number ChiCTR2200055835.Written informed consent was obtained from the parents of each patient.

### Participants

The children with OSAS who were diagnosed and required surgical treatment at Hangzhou First People's Hospital affiliated to Zhejiang University, were selected as study subjects. Inclusion criteria: (1) American Society of Anesthesiologists (ASA) classification of Grade II; (2) Age 3–14 years old; (3) Meeting the diagnostic criteria of the Chinese Guidelines for the Diagnosis and Treatment of Obstructive Sleep Apnea in Children. Exclusion criteria: (1) Children with recent pulmonary infection or respiratory tract infection; (2) Children with cardiopulmonary, hepatic or renal insufficiency; (3) Children who do not meet the observation position during the extubation period after general anesthesia (e.g., unable to maintain a specific head-high lateral position, etc.); (4) Children with mental incompetence (e.g., cerebral palsy).

## Methods

### Determination of sample size

Using G-power (version3.1.9.2) software to calculate the sample size, the main research index of this study was the difference in pulse oxygen saturation of children after extubation. According to the results of the previous pre-test (a total of 90 children were included, among which the mean pulse oxygen saturation after extubation with the supine position was 95.7%, the mean pulse oxygen saturation with the head-high 15°in lateral position was 96.2%, and the mean pulse oxygen saturation with the head-high 15°in lateral position was 96.3%), the setting = 0.05, power = 0.95, 122 children were included in each group. Considering that the dropout rate was 0.2, according to the formula dropout rate = number of dropouts / total sample size, a total of 153 children should be included in each group.

### Randomization

In this study, a total of 459 children undergoing tonsillectomy and adenoidectomy under general anesthesia were recruited, and the children were numbered 1 to 459 in order of surgery, and the random number list generated by Graphpad Prism7 was used for randomization, and finally the A, B, and C corresponding to each number were obtained, and then A, B, and C were sealed in envelopes with corresponding numbers and kept by the study leader. After obtaining written informed consent, the researchers unsealed the envelope to clarify which group the child was assigned to.

The trial was conducted on a single-blind basis (i.e., the participants were unaware of the grouping, and the investigators were aware of the child's grouping). The 459 children after OSAS surgery under general anesthesia were randomly divided into three groups, with 153 children in each group. The three groups were Group A: Head-high 0° in right lateral position; Group B: Head-high 15° in right lateral position; Group C: Head-high 30° in right lateral position.

### Pre-anesthesia preparation

The child was visited one day before surgery and signed an informed consent form for anesthesia. All children fasted for 8 h before surgery and abstained from drinking for 2 h before surgery. After admission, the children were routinely monitored for electrocardiogram (ECG), heart rate (HR), pulse oxygen saturation (SpO_2_), noninvasive blood pressure (NIBP), electroencephalographic bispectral index (BIS), and body temperature (T).

### Anesthesia induction

Before induction of general anesthesia, all children were given 0.01 mg/kg atropine by sedation to reduce airway secretions, and at the beginning of induction of anesthesia, midazolam (0.1 mg/kg), cis-atracurium (0.1 mg/kg), propofol (2 mg/kg), and sufentanil (0.4 μg/kg) were given sequentially under pure oxygen by mask inhalation (later assisted or controlled breathing). 3 to 5 min to be perfected by muscle relaxation and then tracheal intubation was performed under visual laryngoscope. (catheter diameter (with sleeve) = age/4 + 4; catheter insertion depth through the mouth = age/2 + 12 cm) [13]. After auscultation of clear and symmetrical respiratory sounds in both lungs, the cannula was properly fixed and connected to the anesthesia machine to control respiration, ventilation mode: volume-controlled ventilation, inspired oxygen concentration 50%, tidal volume 8-10 ml/kg, minute ventilation volume 100-200 ml/kg, respiratory rate 14-18breaths/min, inspiration-expiration ratio 1:2, peak inspiratory pressure 12-20cmH_2_O (the upper-pressure limit should not exceed the maximum 28cmH_2_O), end-tidal carbon dioxide (ETCO_2_) is maintained at 35-45 mmHg, and respiratory rate and tidal volume are adjusted according to ETCO_2_ [14].

### Anesthesia maintenance

The same surgical approach was used for all children. During the procedure, sevoflurane 1.5% by inhalation, propofol (6–10 mg/kg/h) and remifentanil (0.05–0.1ug/kg/min) were pumped to all children to maintain an electroencephalographic bispectral index (BIS) of 40–60, while the pumping rate of intravenous anesthetic drugs was adjusted according to the BIS values, heart rate and blood pressure of the children.

### Post-operative management

**Fig. 1:**
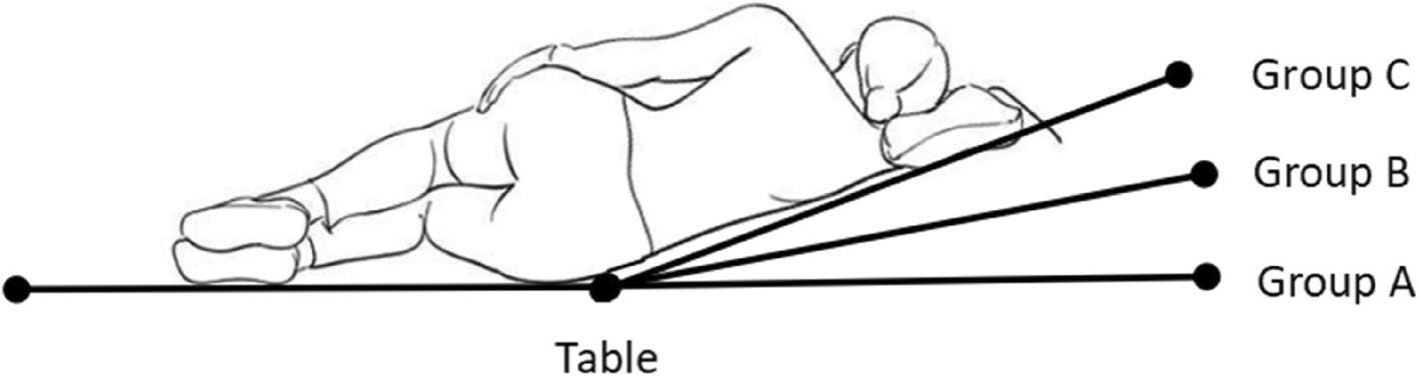
Body position diagram. Note: Group A (Head-high 0° in right lateral position), Group B (Head-high 15° in right lateral position), Group C (Head-high 30° in right lateral position)

After the surgery, all anesthetic drugs were stopped, and the children were taken to the Post Anesthesia Care Unit (PACU) smoothly, and after connecting the ventilator and ECG monitoring, the children were divided into three groups according to the random number method, and aspiration tubes were used to attract secretions from the oropharynx of the children. And then the positions were arranged according to the groups: Group A: Head-high 0° in right lateral position, Group B: Head-high 15° in right lateral position, and Group C: Head-high 30° in right lateral position, as shown in Fig. 1. The tracheal tube was removed when the child regained spontaneous breathing (respiratory rate ≥ 8 breaths/min, tidal volume ≥ 6 mL/kg and SpO_2_ ≥ 95%) and consciousness (able to complete commands such as eye-opening, head raising and hand holding) and when swallowing and coughing reflexes were active.

### Observed parameters

Record the HR, BP, SpO_2_, ETCO_2_, and RR of the child at T0 (at the time of admission to the PACU), T1 (before extubation), T2 (immediately after extubation), T3 (1 min after extubation), T4 (10 min after extubation) and T5 (at the time of discharge from the PACU).

Record the presence of respiratory complications such as choking, laryngospasm, posterior tongue drop, and hypoxemia after extubation at the corresponding time point.

Record the time of awakening and the amount of oral and nasal secretions (g) of the child before extubation.

Record the Sedation-Agitation Scale (SAS) scores of the child at each time point after awakening. The SAS scores [15] was used to assess the agitation of the child, and the scoring criteria were: the child could not be awakened and did not respond to stimuli as 1; the child was very sedated and only responded to somatic stimuli as 2; the child was drowsy and could be awakened as 3; the child was quiet and cooperative and obeyed commands as 4; the child was agitated but could be verbally persuaded as 5; the child was agitated and could not be persuaded as 6; dangerous agitation of the child is 7.

### Statistical analysis

The SPSS 27.0 statistical software was used to statistically analyze the data obtained, and the measures were tested for normality and chi-square, and the normally distributed measures were expressed as mean ± standard deviation (x̅ ± s), and the data were compared among the three groups using one-way ANOVA, and the data with statistically significant differences were further compared between groups using the LSD test. Non-normally distributed measures were expressed as median or interquartile range, and the data were compared using the rank sum test; count data were expressed as rates (%) and compared using the chi-square (χ2) test. *p* < 0.05 was considered statistically significant.

## Results

**Fig. 2:**
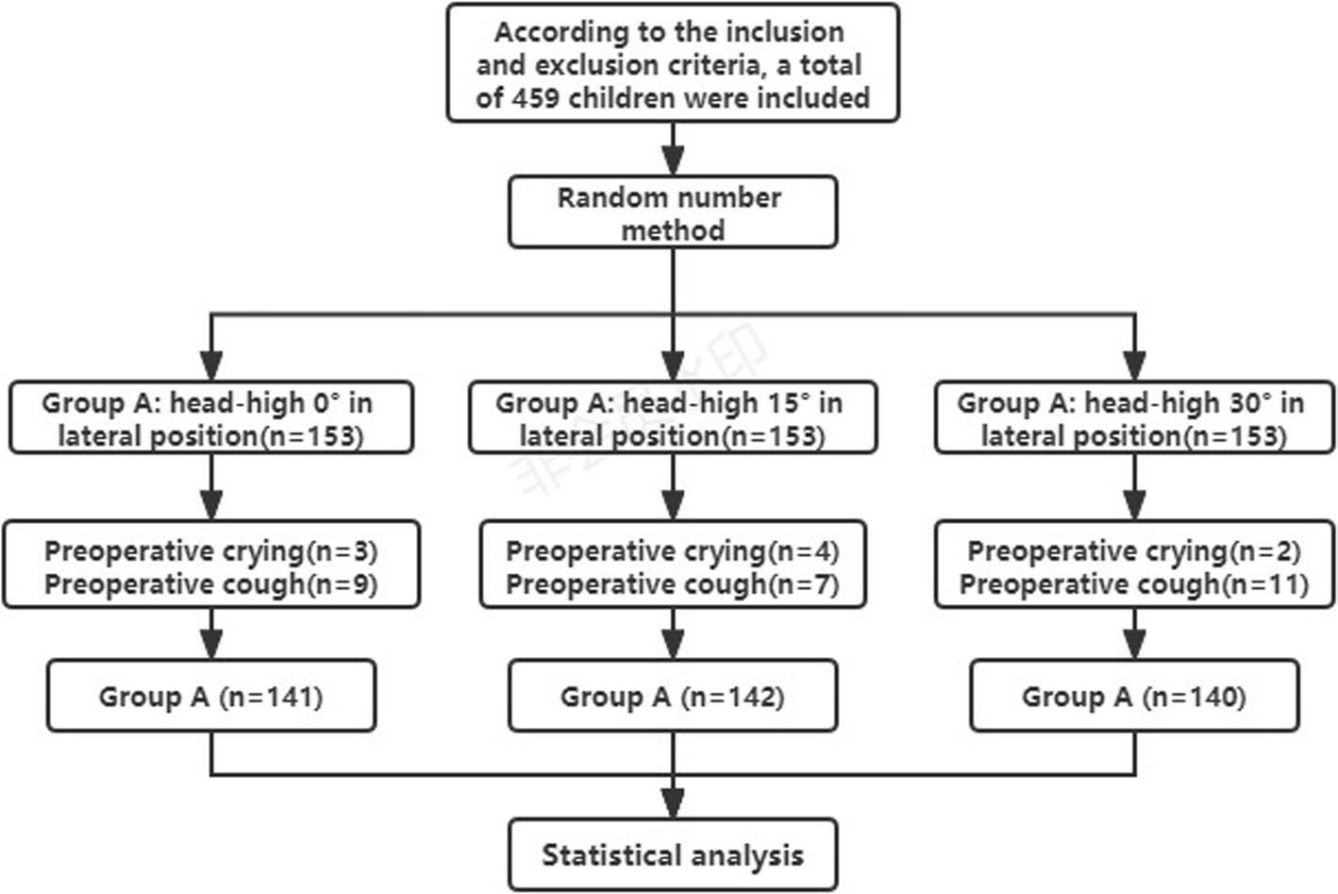
Flow Chart

According to the inclusion and exclusion criteria, a total of 459 children were recruited in this study, 153 in each group. Among them, Group A: 3 children were crying and difficult to persuade before surgery, and 9 children with cough symptoms before surgery were excluded, a total of 141 children were included; Group B: 4 children were crying and difficult to persuade before surgery, and 7 children with cough symptoms before surgery were excluded, a total of 142 children were included; Group C: 2 children were crying and difficult to persuade before surgery, and 11 children with cough symptoms before surgery were excluded, a total of 140 children were included, as shown in Fig. 2.

### Comparison of the general data of the three groups of children

The general conditions of the three groups of children such as age, height, weight, and gender were not significantly different (all *P* > 0.05) and were comparable, as shown in Table 1.

**Table 1:** Comparison of general data among the three groups

	Gender(M/F)	Age (years)	Height(cm)	Weight (kg)
Group A(*n* = 141)	92/49	5.9 $\pm$ 2.0	116.4 $\pm$ 14.5	22.5 $\pm$ 8.1
Group B(*n* = 142)	91/51	6.0 $\pm$ 2.1	116.6 $\pm$ 15.3	22.5 $\pm$ 8.4
Group C(*n* = 140)	92/48	6.1 $\pm$ 2.0	117.5 $\pm$ 14.1	22.6 $\pm$ 8.1
*P*	0.690	0.616	0.791	0.899

Data are expressed as mean ± standard deviation. Group A (head-high 0° in lateral position), Group B (head-high 15° in lateral position), Group C (head-high 30° in lateral position)

### Comparison of HR, SpO_2_, ETCO_***2***_ and RR at T0 in three groups of children

The comparisons of HR, SpO_2_, ETCO_2_, and RR at the time of admission to the PACU(T0) were not significantly different (all *P* > 0.05) and were comparable, as shown in Table 2.

**Table 2:** Comparison of HR, SpO_2_, ETCO_2_, and RR at T0 in the three groups of children

	HR	SpO_2_	ETCO_2_	RR
Group A(*n* = 141)	93.5 $\pm$ 16.6	99.2 $\pm$ 1.0	35.1 $\pm$ 4.7	16.7 $\pm$ 1.5
Group B(*n* = 140)	93.5 $\pm$ 16.2	99.2 $\pm$ 1.0	34.5 $\pm$ 4.9	16.6 $\pm$ 1.4
Group C(*n* = 142)	93.2 $\pm$ 16.4	99.4 $\pm$ 1.0	34.7 $\pm$ 5.6	16.6 $\pm$ 1.6
*P*	0.883	0.386	0.754	0.272

Data are expressed as mean ± standard deviation. Group A (head-high 0° in lateral position), Group B (head-high 15° in lateral position), Group C (head-high 30° in lateral position). Abbreviations: *HR* Heart Rate, *SpO*_*2*_ Pulse Oxygen Saturation, *ETCO*_*2*_ End-Respiratory Carbon Dioxide, *RR* Respiratory Rate

### Comparison of SpO_2_ in three groups of children at different time periods

**Fig. 3:**
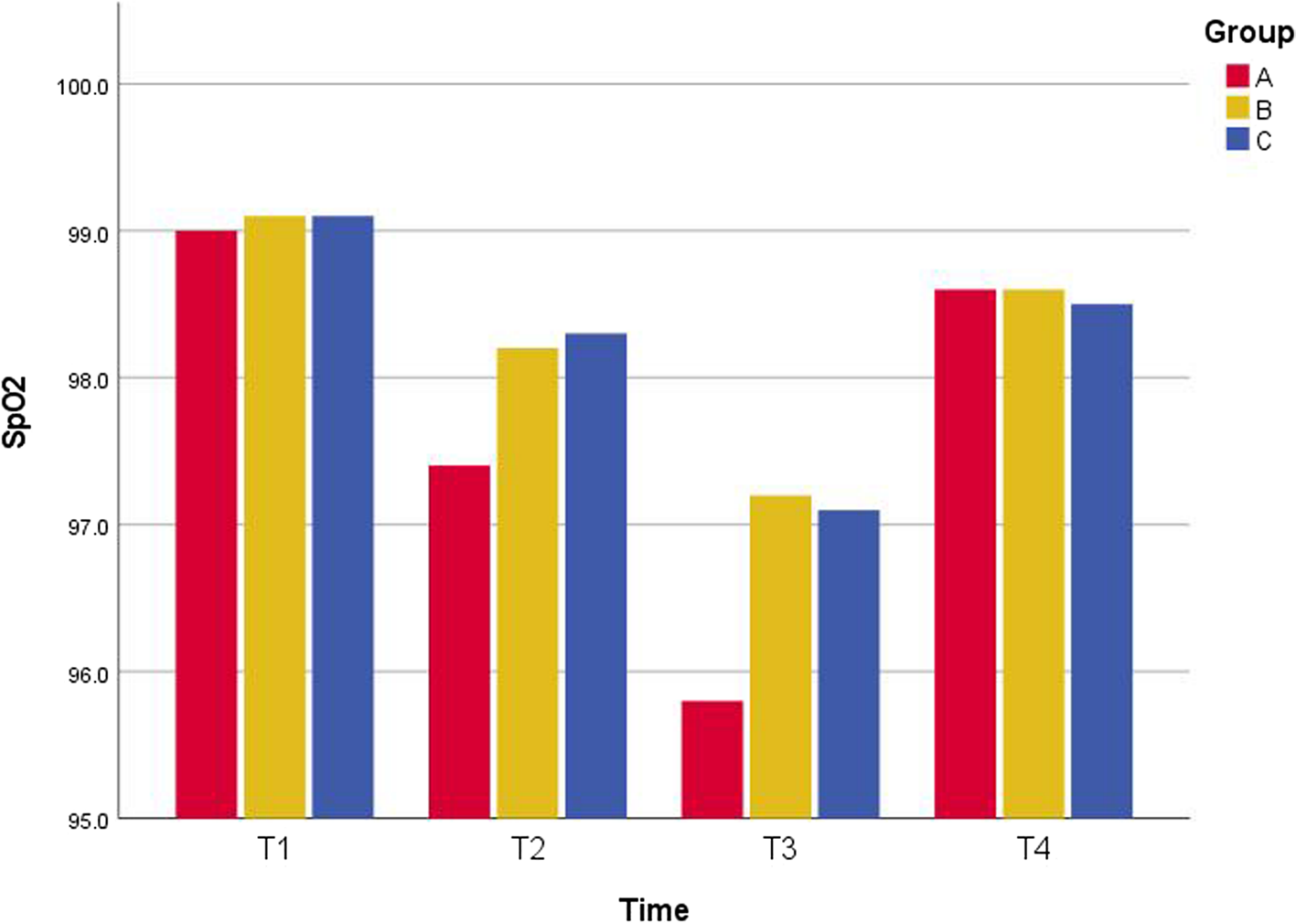
Comparison of SpO2 in the three groups of children at different time periods, Note: Data are expressed as mean. Group A (head-high 0° in lateral position), Group B (head-high 15° in lateral position), Group C (head-high 30° in lateral position), T1 (before extubation), T2 (immediately after extubation), T3 (1 min after extubation), T4 (10 min after extubation). Abbreviations: SpO2 (Pulse Oxygen Saturation)

The mean values of SpO_2_ in children in Group A at T2 and T3 were significantly lower than those in Group B and Group C, and the differences were statistically significant (all *P* < 0.05), while no significant differences were seen in the mean values of SpO_2_ in the three groups at T1 and T4 (all *P* > 0.05). And the mean values of SpO_2_ in Group B and Group C did not show significant differences and were not statistically significant (all *P* > 0.05), as shown in Tables 3, 4, and Fig. 3.

**Table 3:** Comparison of SpO_2_ in the three groups of children at different time periods

	T1	T2	T3	T4
Group A(*n* = 141)	99.0 $\pm$ 1.0	97.4 $\pm$ 2.3	95.8 $\pm$ 3.8	98.6 $\pm$ 1.2
Group B(*n* = 140)	99.1 $\pm$ 0.8	98.2 $\pm$ 1.7	97.2 $\pm$ 2.8	98.6 $\pm$ 1.3
Group C(*n* = 142)	99.1 $\pm$ 0.7	98.3 $\pm$ 1.5	97.1 $\pm$ 2.6	98.5 $\pm$ 1.2
*P*	0.162	< 0.001^a^	< 0.001^a^	0.660

Data are expressed as mean ± standard deviation. Group A (head-high 0° in lateral position), Group B (head-high 15° in lateral position), Group C (head-high 30° in lateral position), T1 (before extubation), T2 (immediately after extubation), T3 (1 min after extubation), T4 (10 min after extubation)

^a^indicates a statistically significant difference

**Table 4:** Comparison between the three groups of children with SpO_2_ at T2 and T3

Time	Groups	*P*
T2	AB	< 0.001^a^
	AC	< 0.001^a^
	BC	0.681
T3	AB	< 0.001^a^
	AC	< 0.001^a^
	BC	0.634

Group A (head-high 0° in lateral position), Group B (head-high 15° in lateral position), Group C (head-high 30° in lateral position), T2 (immediately after extubation), T3 (1 min after extubation)

^a^indicates a statistically significant difference

### Comparison of SAS in three groups of children at different time periods

**Fig. 4:**
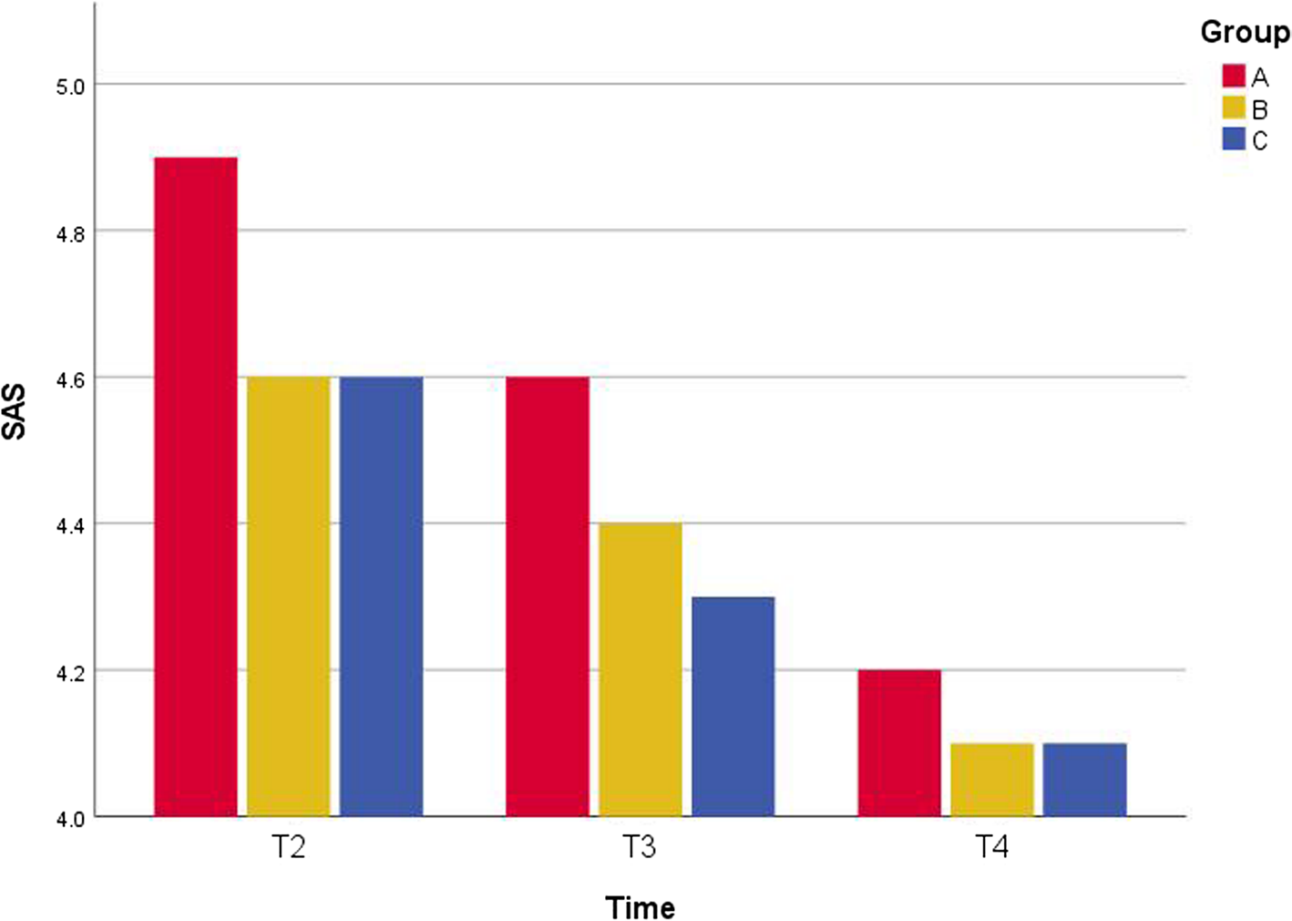
Comparison of SAS in the three groups of children at different time periods. Note: Data are expressed as mean. Group A (head-high 0° in lateral position), Group B (head-high 15° in lateral position), Group C (head-high 30° in lateral position), T2 (immediately after extubation), T3 (1 min after extubation), T4 (10 min after extubation). Abbreviations: SAS (sedation-restlessness scale)

The mean values of SAS of children in Group A at T2 and T3 were significantly higher than those in Group B and Group C, and the differences were statistically significant (all *P* < 0.05). While the differences in SAS between Group B and Group C were not statistically significant (all *P* > 0.05), as shown in Table 5, 6, and Fig. 4.

**Table 5:** Comparison of SAS in the three groups of children at different time periods

	T2	T3	T4
Group A(*n* = 141)	4.9 $\pm$ 0.9	4.6 $\pm$ 0.9	4.2 $\pm$ 0.5
Group B(*n* = 140)	4.6 $\pm$ 0.8	4.4 $\pm$ 0.7	4.1 $\pm$ 0.4
Group C(*n* = 142)	4.6 $\pm$ 0.8	4.3 $\pm$ 0.6	4.1 $\pm$ 0.3
*P*	< 0.001^a^	< 0.001^a^	0.180

Data are expressed as mean ± standard deviation. Group A (head-high 0° in lateral position), Group B (head-high 15° in lateral position), Group C (head-high 30° in lateral position), T2 (immediately after extubation), T3 (1 min after extubation), T4 (10 min after extubation). Abbreviations: *SAS* sedation-restlessness scale

^a^indicates a statistically significant difference

**Table 6:** Comparison of SAS at T2 and T3 in the three groups of children

Time	Groups	*P*
T2	AB	0.003^a^
	AC	< 0.001^a^
	BC	0.425
T3	AB	0.001^a^
	AC	< 0.001^a^
	BC	0.723

Group A (head-high 0° in lateral position), Group B (head-high 15° in lateral position), Group C (head-high 30° in lateral position), T2 (immediately after extubation), T3 (1 min after extubation). Abbreviations: *SAS* sedation-restlessness scale

^a^indicates a statistically significant difference

### Comparison of choking and coughing in three groups of children at different times

The response of children in Group A with choking and coughing at T2 and T3 was higher than those in Group B and Group C, and the difference was statistically significant (all *P* < 0.05). But the difference was not statistically significant compared with Group B and Group C (all *P* > 0.05), as shown in Tables 7 and 8.

**Table 7:** Comparison of choking and coughing in the three groups of children at different times (%)

	T2	T3
A	85/141(60.3%)	40/141(28.4%)
B	66/142(46.5%)	21/142(14.8%)
C	57/140(40.7%)	11/140(7.9%)
*P*	0.004^*^	< 0.001^a^

Data are expressed as rates (%). Group A (head-high 0° in lateral position), Group B (head-high 15° in lateral position), Group C (head-high 30° in lateral position), T2 (immediately after extubation), T3 (1 min after extubation)

^a^indicates a statistically significant difference

**Table 8:** Comparison between groups of children with choking cough at T2 and T3

Time	Groups	*P*
T2	AB	0.025^a^
	AC	0.001^a^
	BC	0.320
T3	AB	0.002^a^
	AC	< 0.001^a^
	BC	0.113

Group A (head-high 0° in lateral position), Group B (head-high 15° in lateral position), Group C (head-high 30° in lateral position), T2 (immediately after extubation), T3 (1 min after extubation)

^a^indicates a statistically significant difference

### Comparison of the HR in three groups of children at different time periods

The comparison of the heart rates of the three groups of children at each time period was not significantly different and not statistically significant (all *P* > 0.05), as shown in Table 9.

**Table 9:** Comparison of the HR of the children at different time periods

	T1	T2	T3	T4
Group A(*n* = 141)	111.6 $\pm$ 18.5	119.6 $\pm$ 18.4	113.9 $\pm$ 19.9	107.5 $\pm$ 18.4
Group B(*n* = 142)	110.6 $\pm$ 18.1	118.1 $\pm$ 17.9	112.8 $\pm$ 18.2	107.7 $\pm$ 17.4
Group C(*n* = 140)	109.1 $\pm$ 20.0	116.7 $\pm$ 19.2	111.6 $\pm$ 19.4	106.8 $\pm$ 17.4
*P*	0.525	0.388	0.579	0.901

Data are expressed as mean ± standard deviation. Group A (head-high 0° in lateral position), Group B (head-high 15° in lateral position), Group C (head-high 30° in lateral position), T1 (before extubation), T2 (immediately after extubation), T3 (1 min after extubation), T4 (10 min after extubation)

Abbreviations: *HR* Heart Rate

### Comparison of the outflow of oral and nasal secretions during the awakening period in three groups of children

There was no significant difference in the comparison of the outflow of oral and nasal secretions among the three groups of children, which was not statistically significant (all *P* > 0.05), as shown in Table 10.

**Table 10:** Comparison of the weight of oral and nasal secretions during the awakening period

	Secretions(g)
Group A(*n* = 141)	2.1 $\pm$ 1.7
Group B(*n* = 142)	2.1 $\pm$.1.5
Group C(*n* = 140)	2.2 $\pm$ 1.6
*P*	0.162

Data are expressed as mean ± standard deviation. Group A (head-high 0° in lateral position), Group B (head-high 15° in lateral position), Group C (head-high 30° in lateral position)

### Comparison of awakening time in three groups of children

There was no significant difference in the comparison of awakening time among the three groups of children, which was not statistically significant (all *P* > 0.05), as shown in Table 11.

**Table 11:** Comparison of awakening times of children in the three groups

	Awakening Time(min)
Group A(*n* = 141)	22.8 $\pm$ 9.5
Group B(*n* = 142)	22.6 $\pm$ 9.1
Group C(*n* = 140)	24.9 $\pm$ 10.2
*P*	0.177

Data are expressed as mean ± standard deviation. Group A (head-high 0° in lateral position), Group B (head-high 15° in lateral position), Group C (head-high 30° in lateral position)

## Discussion

In this study, we finally analyzed the data of 423 children with different head-high lateral positions to drain the secretions, blood and surgical irrigation fluid from the pharynx of the children through the positions in order to reduce the occurrence of adverse events such as respiratory complications and post-operative agitation after extubation. In our study, a head-high 15° lateral position and a head-high 30° lateral position during post-operative extubation in children with OSAS significantly reduced the risk of post-extubation choking and hypoxia in children. In addition, the occurrence of agitation after awakening of the children was significantly reduced.

The clinical morbidity of OSAS in children is high and its surgical treatment is based on the removal of enlarged tonsils and adenoids. The smooth resuscitation period is crucial for the safety of the children's life during extubation, especially in the post-operative period for children with OSAS. The vital signs of the children should be closely monitored after extubation. Post-extubation complications include bronchospasm, severe cough, laryngospasm, decreased oxygen saturation, laryngeal edema, pulmonary atelectasis, pulmonary edema, apnea and hemodynamic instability [16], among which cough, decreased oxygen saturation and laryngospasm are the most common post-extubation complications [17]. In this study, the main indexes included the pulse oxygen saturation of children in six time periods, T0 (at the time of admission to PACU), T1 (before extubation), T2 (immediately after extubation), T3 (1 min after extubation), T4 (10 min after extubation), and T5 (at the time of discharge from PACU), as well as the sedation-agitation scale (SAS) score, the amount of oral and nasal discharge, and the presence of respiratory complications such as choking, laryngospasm, and hypoxemia after extubation. After the surgery, the children's anesthesia gradually decreased, but the tracheal tube inserted in the trachea as a foreign body in the airway, easy to irritate the endotracheal mucosa, causing the children to cough and agitation. The incidence of agitation during the awakening period of general anesthesia is often sudden and is about 5.3% in adult patients, but the incidence of agitation in pediatric patients has been reported in the paper to be as high as l0-67% [18], and the incidence may be higher in children with OSAS undergoing tonsillectomy and adenoidectomy because of the specificity of the population and the surgical site. In addition, children may struggle and become agitated during extubation, and some may even suddenly remove the tracheal tube on their own, which may not only increase surgical site injury and bleeding, but also lead to aspiration, laryngospasm, and airway obstruction [19], and even cause posterior dislocation of the arytenoid cartilage and serious complications and injuries related to extubation, such as postoperative choking and hoarseness.

In this study, the lateral position was combined with the head-high supine position and divided into head-high 0°, head-high 15° and head-high 30° lateral position for extubation. It has been demonstrated that after tonsil/adenoids removal surgery under general anesthesia in pediatric patients, the SpO_2_ after extubation in children who adopted the lateral position (mean value 98.3%) was better than that after extubation in the supine position (mean value 96.8%) [9], which is also in accordance with our study design. In our study, the mean SpO_2_ values immediately after extubation (98.2% and 98.3%, respectively) and 1 min after extubation (97.2% and 97.1%, respectively) of children with OSAS were higher in the head-high 15° lateral position and in the head-high 30° lateral position during general anesthesia awakening after tonsillectomy and adenoidectomy than in the supine (head-high 0°) position. The mean SpO_2_ values immediately after extubation and 1 min after extubation were 97.4% and 95.8% in children in the lateral position, respectively. This confirms that children with OSAS are more able to maintain a relatively stable pulse oxygen saturation in the head-high lateral position after tonsillectomy and adenoidectomy compared to the lateral position. It has been shown that patients with general anesthesia are more comfortable in an appropriate head-high position (15° to 30°) after awakening. Combined with the analysis of the results of this study, this may be because in the head-high position, the children's diaphragm decreases and pulmonary compliance increases, which is more conducive to the children's breathing and reduces the work done by the respiratory muscles, and also provides a more comfortable position.

In addition, the incidence of choking (46.5% and 40.7%, respectively) and SAS score (4.6 and 4.6, respectively) in children with head-high 15° lateral position and head-high 30° lateral position were also significantly lower than the incidence of choking (60.3%) and SAS score (4.9) in children with head-high 0° lateral position. This may be due to the fact that extubation in the lateral position allows secretions and blood to flow automatically from the mouth and nose, reducing their irritation to the children's pharynx [20]. The head-high position is more conducive to the children's swallowing, and some of the secretions, blood and irrigation fluid in the children's pharynx flow out through the mouth and nose, while the other part can flow into the stomach along the esophagus, reducing the fluid in the children's pharynx. Therefore, during extubation, as the fluid in the children's pharynx is reduced, the irritation to the pharynx and even the trachea is reduced, and the children's choking and coughing reactions are reduced, so the agitation of the children during awakening is also reduced. Although postoperative extubation in the lying position is the most mainstream method, the secretions in the pharynx of children in the lying position do not flow out easily, and when the suction tube is used frequently to attract secretions after surgery, however, it is difficult to ensure that all the secretions are removed because the suction tube is used under non-direct vision, and the suction tube goes in and out of the pharynx several times to attract, which may cause bleeding in the traumatic surface of the tonsil fossa and adenoid fossa [9], and also causes discomfort in the pharynx of children and increases the risk of respiratory reflux aspiration. If the suction tube is used during shallow anesthesia, it is more likely to stimulate the children's larynx and cause complications such as laryngospasm, violent choking and awakening agitation [21, 22]. This shows that we have combined the head-high position with the lateral position, and it is very correct to use the head-high lateral position for extubation in children with OSAS after surgery.

However, in our study, there was no significant difference between the two groups in either the incidence of airway complications such as choking and laryngospasm or the incidence of adverse events such as postoperative agitation in the head-high 15° lateral position compared with the head-high 30° lateral position (all *P* > 0.05), showing that under the conditions of this clinical trial study, a higher head-high position (head-high more than 30°) compared with the head-high 15° lateral position did not further reduce the occurrence of complications during extubation in children with OSAS. This may be due to the fact that when the children are in the head-high 15° lateral position, the secretions and blood from the pharynx have flowed out along the mouth and nose or into the stomach along the esophagus. Therefore, when the children are in the head-high 30° lateral position, the fluid in the children's throat cannot be further reduced, and the adverse reactions such as choking and agitation cannot be further reduced. In addition, although there was no statistical difference in the occurrence of respiratory complications and adverse events during extubation between the head-high 15° lateral position and the head-high 30° lateral position, we observed that the lateral flexion angle of the spine was larger in the head-high 30° lateral position, and maintaining this position for a long time may cause some children's somatic discomfort. Therefore, this study recommends a head-high 15° lateral position for extubation.

This study has some limitations. Firstly, limited by the conditions, we did not use double-blind RCT, which may have some subjective error; Secondly, we selected children aged 3–14 years, and there are some differences in the cognitive levels of the children, and the results obtained may be more accurate if the conditions allow for grouping by different age groups.

## Conclusions

Firstly, during general anesthesia awakening after tonsillectomy and adenoidectomy of children with OSAS, the use of a head-high lateral position of 15° or greater is effective in reducing the incidence of adverse events such as choking and agitation after extubation, as well as reducing the degree of decrease in pulse oxygen saturation after extubation compared with a flat lateral position.

Secondly, there was no significant difference in post-extubation complications (e.g., choking, agitation, decreased oxygen saturation, etc.) during general anesthesia awakening after tonsillectomy and adenoidectomy of children with OSAS in the head-high 15° lateral position compared to the head-high 30° lateral position.Considering the comfort of the children and the ease of position placement, this study recommends a head-high 15° lateral position as the ideal position for extubation during general anesthesia awakening in children with OSAS.

## Data Availability

The datasets used and/or analysed during the current study are available from the corresponding author on reasonable request.

## Abbreviations

BISE: Lectroencephalographic Bispectral Index
ECG: Electrocardiogram
ETCO_2_: End-tidal Carbon Dioxide
HR: Heart Rate
NIBP: Non-invasive Blood Pressure
OSAS: Obstructive Sleep Apnea Syndrome
PACU: Post Anesthesia Care Unit
RR: Respiratory Rate
SAS: Sedation-Agitation Scale
SpO_2_: Pulse Oxygen Saturation
